# Early Botulinum Toxin Type A Injection for Post-Stroke Spasticity: A Longitudinal Cohort Study

**DOI:** 10.3390/toxins13060374

**Published:** 2021-05-24

**Authors:** Alessandro Picelli, Andrea Santamato, Michela Cosma, Alessio Baricich, Carmelo Chisari, Marzia Millevolte, Cristina Del Prete, Ilenia Mazzù, Paolo Girardi, Nicola Smania

**Affiliations:** 1 Department of Neurosciences, Biomedicine and Movement Sciences, University of Verona, 37134 Verona, Italy; alessandro.picelli@univr.it; 2 Department of Clinical and Experimental Medicine, University of Foggia, 71122 Foggia, Italy; andrea.santamato@unifg.it; 3 Neuroscience and Rehabilitation Department, Ferrara University Hospital, 44124 Ferrara, Italy; m.cosma@ospfe.it; 4 Department of Health Sciences, University of Piemonte Orientale, 28100 Novara, Italy; alessio.baricich@med.uniupo.it; 5 Department of Translational Research on New Technologies in Medicine and Surgery, University of Pisa, 56126 Pisa, Italy; carmelo.chisari@unipi.it; 6 Department of Neuroscience, Ancona University Hospital, 60123 Ancona, Italy; marzia.millevolte@ospedaliriuniti.marche.it; 7 G. Panico Hospital, 73039 Tricase, Italy; cridelprete@tin.it; 8 IRCCS Santa Lucia Foundation, 00179 Rome, Italy; i.mazzu@hsantalucia.it; 9 Department of Developmental Psychology and Socialisation, University of Padua, 35121 Padua, Italy; paolo.girardi@unipd.it

**Keywords:** botulinum toxins, muscle spasticity, rehabilitation, stroke, therapeutics

## Abstract

Early management of spasticity may improve stroke outcome. Botulinum toxin type A (BoNT-A) is recommended treatment for post-stroke spasticity (PSS). However, it is usually administered in the chronic phase of stroke. Our aim was to determine whether the length of time between stroke onset and initial BoNT-A injection has an effect on outcomes after PSS treatment. This multicenter, longitudinal, cohort study included stroke patients (time since onset <12 months) with PSS who received BoNT-A for the first time according to routine practice. The main outcome was the modified Ashworth scale (MAS). Patients were evaluated before BoNT-A injection and then at 4, 12, and 24 weeks of follow-up. Eighty-three patients with PSS were enrolled. MAS showed a significant decrease in PSS at 4 and 12 weeks but not at 24 weeks after treatment. Among the patients with a time between stroke onset and BoNT-A injection >90 days, the MAS were higher at 4 and 12 weeks than at 24 weeks compared to those injected ≤90 days since stroke. Our findings suggest that BoNT-A treatment for PSS should be initiated within 3 months after stroke onset in order to obtain a greater reduction in muscle tone at 1 and 3 months afterwards.

## 1. Introduction

Damage to the sensorimotor networks and descending tracts results in the upper motor neuron syndrome (UMNS) [1]. Spasticity is a positive symptom of UMNS and has been defined as “a motor disorder characterized by a velocity-dependent increase in tonic stretch reflexes (muscle tone) with exaggerated tendon jerks, resulting from hyperexcitability of the stretch reflex” [2]. The prevalence of spasticity after first-ever stroke is 4–27% within the first 6 weeks, 19% at 3 months, 21.7–42.6% between 4 and 6 months, and 17–38% at 12 months from onset [3,4,5].

Spasticity may impact on the disability of stroke patients [6,7]. Prediction of post-stroke spasticity (PSS) can help to prevent (if possible) its onset, slow or limit its progression [8]. Early detection of PSS can improve the long-term outcome of stroke patients [3,8,9].

Botulinum toxin type A (BoNT-A) is a recommended treatment for PSS [10,11,12]. Despite the growing evidence for early treatment of PSS [13,14,15], BoNT-A is usually administered to chronic stroke patients in routine clinical practice [16,17], probably because of the variable prevalence of PSS during the first year after onset and because the published literature about BoNT-A for PSS reports mainly on treatment during the chronic phase [3]. With this study, we wanted to determine whether the time between stroke onset and initial BoNT-A injection had an effect on outcomes after treatment for PSS in routine clinical practice.

## 2. Results

The study sample was 83 patients with PSS, 52 of whom were men (62.7%). The average age at stroke was 63.9 years (±12.5 years). The time between stroke onset and initial BoNT-A injection was, on average, 136.1 days (±95.1 days). The etiology of stroke was ischemic (69.9%) with a slight prevalence of right-sided lesion (54.2%). BoNT-A treatment was administered to inpatients (48.2%), outpatients (39.8%), and day-hospital patients (12.0%). The injection technique was ultrasound-guided (65.1%), manual needle placement (21.7%), and electrical stimulation (13.3%). The most used dilution rate was 2 mL per vial (67.5%). There was no significant difference between other characteristics and patient sex (Table 1).

As to the volitional activity of antagonist muscles measured on the Medical Research Council (MRC) scale, the wrist and finger extensors showed the lower profile (median 0, interquartile range (IQR) 2), while the muscle groups with the best profile were the hip extensors (median 3, IQR 1), the knee (median 3, IQR 2), and the knee flexors (median 3, IQR 2). Differences in elbow extensor functionality (*p* = 0.04) and ankle pronators (*p* = 0.03) between the sexes were noted (Table 2).

Table 3 presents the Fugl-Meyer assessment (FMA) scores by follow-up time: the upward trend was significant for the upper limb (Kendall trend test, *p* = 0.001), for the wrist (*p* = 0.023) in particular, speed coordination (*p* = 0.045), motor function (*p* = 0.016), and pain (*p* = 0.007); there was a significant time variation for the lower limb (*p* = 0.002), which was more pronounced for speed coordination (*p* < 0.001), motor function (*p* < 0.001), and sensation (*p* < 0.045).

**Figure 1:**
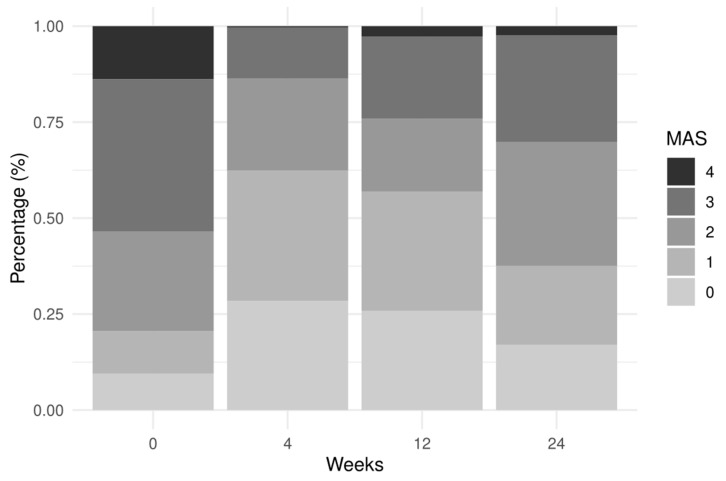
Frequency distribution of MAS and MRS by follow-up time.

The Modified Ashworth Scale (MAS) scores were decreased at week 4 after the initial BoNT-A injection, with a progressively smaller decrease at 12 and 24. Conversely, there was a decrease in the Modified Rankin Scale (MRS) scores through to the end of follow-up (Figure 1).

**Figure 2:**
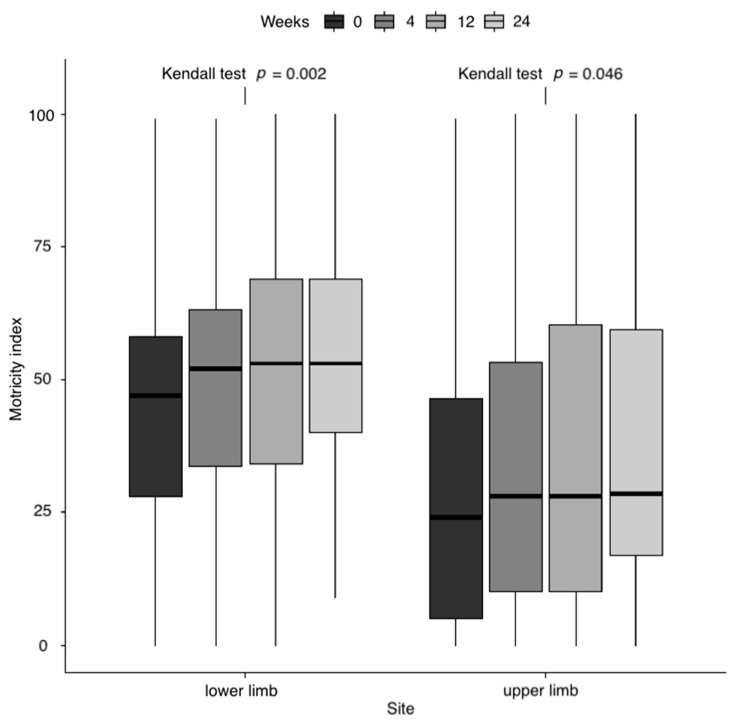
Distribution of MI values by follow-up time and site. *p*-Value resulting from the Kendall correlation test between MI and weeks was reported by site.

There was a progressive increase in the Motricity Index (MI) over time for the lower limb (Kendall test, *p* = 0.002), while the increase for the upper limb was less pronounced (*p* = 0.046) (Figure 2).

A total of 1167 MAS measurements of some muscle groups were performed, most often of the wrist flexors (*n* = 204, 17.2%), the finger flexors (*n* = 192, 16.2%), and the elbow flexors (*n* = 184, 15.5%). There was a significant difference between the group of treated muscles and patient sex (*p* = 0.003) (see Table 4).

The coefficients estimated by the cumulative logistic model showed a reduction in MAS score at 4 (odds ratio (OR) 0.05, 95% confidence interval (CI) 0.03–0.09), at 12 (OR 0.06, 95% CI 0.03–0.09), and at 24 weeks (OR 0.24, 95% CI 0.14–0.41) compared to baseline (T0), with a more pronounced decrease at 4 weeks. The MAS score was lower in patients aged >70 years than in patients <60 years (*p* = 0.013). Vial dilution was inversely correlated with MAS score (OR 0.12 at + 1 mL increase, 95% CI 0.05–0.30). A significant interaction (ANOVA, *p* = 0.022) was found between the time of follow-up and that elapsed between stroke onset and BoNT-A injection (TSO): among the patients with a TSO >90 days, MAS scores were higher at 4 weeks (OR 2.05, 95% CI 1.02–4.13) and 12 weeks (OR 2.87, 95% CI 1.46–5.65) than at 24 weeks compared to the TSO category (≤90 days) (see Table 5). No significant interaction was found for the treatment regimen (ANOVA, *p* = 0.29).

## 3. Discussion

Clinical debate surrounds the use of BoNT-A as early intervention for PSS to prevent more severe muscle hypertonia and muscle contracture [13]. To date, however, there is no consensus on the definition of early treatment (BoNT-A injection within 3 months or within 6 months after stroke onset?); and the current literature reports mainly about BoNT-A injection in patients with PSS at more than 6 months after stroke [13,18]. With this longitudinal cohort study, we wanted to determine whether the time between stroke onset and initial BoNT-A injection had an effect on the outcome of naïve patients with PSS in real-life clinical practice. Our main finding was that BoNT-A treatment performed within 3 months since stroke onset in naïve patients with PSS can achieve the maximum effect on muscle tone (as measured on the MAS) at 1 and 3 months of follow-up. This observation is shared by a metanalysis of six randomized controlled trials conducted by Rosales and colleagues, who reported on the beneficial effects of BoNT-A treatment on PSS within 3 months after onset [13]. However, the studies in their metanalysis involved patients at 2 to 12 weeks after stroke, with placebo (five studies) or rehabilitation alone (one study) as the control. Furthermore, the metanalysis included three studies about upper-limb PSS and three about lower-limb PSS [13]. No study included in this metanalysis dealt with PSS involving both the upper and the lower limb (which is the most common presentation) [19].

Our findings suggest that (upper- and/or lower-limb) PSS should be treated within 3 months after onset rather than later so as to obtain a greater reduction in muscle tone at 1 and 3 months afterwards. This information may be useful for planning rehabilitation of stroke patients. In our view, it is also a strong point of our study in comparison to the later double-blind, randomized placebo-controlled trial (the ONTIME study) by Rosales and collaborators, who found positive effects with a fixed dose (500 U reconstructed with 2.5 mL of 0.9% saline) of AbobotulinumtoxinA injected 2–12 weeks after stroke [20]. The authors concluded that their study suggests an optimal time for PSS management, though they did not compare different time points for PSS treatment with BoNT-A (all patients were injected with BoNT-A or placebo between 2 and 12 weeks after stroke) to define which could be optimal. They reported on a delayed time for re-injection (about 150 days) after early BoNT-A treatment, which is probably related to both the time of injection after stroke and the BoNT-A preparation injected (i.e., AbobotulinutoxinA) [21]. Conversely, we investigated the role of time between stroke onset and BoNT-A injection in a real-life, daily practice, setting, considering different time points in order to identify the optimal one for PSS treatment based on its outcome in naïve patients.

A study published by Wissel and colleagues in 2020 evaluated the real-world effectiveness of AbobotulinumtoxinA on the evolution of spasticity in 303 patients with upper-limb PSS according to the time from stroke to the start of BoNT- A treatment [18]. They observed trends for early versus late BoNT-A treatment of PSS but no strong evidence for a greater benefit of early versus late initiation of BoNT-A on muscle tone (MAS scores). Our study shared a similar main aim; however, we found a clear benefit of early initial BoNT-A treatment of PSS (<90 days since stroke onset) versus late initiation of treatment (>3 months and <12 months since stroke). The discrepancy compared to our findings likely stems from the difference in mean time points as defined by Wissel and colleagues (early start <7 months; late start >36 months and <443 months) [18]. Furthermore, they enrolled naïve patients and patients already under BoNT-A treatment for upper-limb PSS and injected only with AbobotulinumtoxinA. Conversely, we included only naïve patients with upper- and/or lower-limb PSS injected with all BoNT-A preparations. This detail further supports our findings that prior exposure to BoNT-A therapy may be a confounder [18].

### Limitations

As to the limitations of our study, the main one is the absence of measurements about treatment goal attainment (e.g., the Goal Attainment Scale) and patient satisfaction with treatment (that could have provided their perspective on PSS management). Second, no blinded outcome assessment was done. Even if this reflects real-life clinical practice, it might represent a potential bias. In order to limit the potential for bias due to unblinded assessment, we tried to ensure an objective (as possible) evaluation by providing the assessors with some training and a detailed manual on the scales used for clinical assessment before study start. Third, because we did not examine BoNT-A re-injection, we have no information about whether it can be delayed because of treatment timing. Fourth, standardized injection techniques and dilutions were not used (see Table 1). Even if this is in line with the observational design of this study, it might represent a limit for reading and interpreting our findings. Fifth concomitant rehabilitation therapies were allowed in agreement with local guidelines and clinical practice. Nonetheless, it is plausible that such approaches vary in type and intensity depending on the phase of stroke (for example, rehabilitation was probably more intensive in inpatients with early subacute stroke than in outpatients with chronic stroke). While this might have influenced our results, it reflects real-life clinical practice with subacute and/or chronic in/outpatients with special needs.

## 4. Conclusions

This multicenter, longitudinal, cohort study suggests that BoNT-A treatment for PSS should be initiated within 3 months after stroke onset in order to obtain a greater reduction in muscle tone at 1 and 3 months afterwards. However, our findings need to be further confirmed (e.g., by means of randomized controlled trials); as well, the issue of early BoNT-A treatment for PSS needs to be further investigated in the future. In our view, further research should focus on (active and passive) functional goals for early intervention and other implications of early BoNT-A treatment of PSS, such as prevention of contracture, compliance to rehabilitation programs and cost analysis. Furthermore, future studies should investigate the role of preventive BoNT-A treatment (i.e., before any development of PSS) in comparison with later injection (e.g., when clinically relevant PSS arises).

## 5. Materials and Methods

This multicenter, open-label, longitudinal, cohort study involved inpatients and outpatients with PSS enrolled between June 2015 and December 2018 at eight university and clinical hospitals throughout Italy. The reporting of study findings follows the STrengthening the Reporting of OBservational studies in Epidemiology (STROBE) criteria [22].

Inclusion criteria were: age ≥18 years; first-ever unilateral ischemic or hemorrhagic stroke (as documented by a computerized tomography scan or magnetic resonance imaging; subarachnoid hemorrhage excluded); time since stroke onset <12 months; PSS ≥ 1+/4 on the MAS involving the affected limb with volitional activity of the antagonist muscles graded ≤2/5 on the MRC scale [23,24]; no previous treatment of PSS with BoNT-A (naïve patients); no other antispastic medications (including muscle relaxants). Exclusion criteria were: participation in other trials; fixed contractures (muscle tone graded 4/4 on the MAS) or bony deformities of the affected limbs; previous neurolytic (phenolization/alcoholization) or surgical treatment for PSS; other neurologic or orthopedic conditions involving the affected limbs. Eligible patients received BoNT-A injection and stroke rehabilitation according to local regulatory and guidelines [25,26].

Written, informed consent was obtained for participation in the study, which was carried out according to the tenets of the Declaration of Helsinki and approved (5 March 2015; approval code 392CESC) by the local ethics committee (Comitato Etico per la Sperimentazione Clinica delle Province di Verona e Rovigo). Clinical Trial Registration-URL: http://www.clinicaltrials.gov, accessed on 23 May 2021 (Unique identifier NCT04404868).

Patients were evaluated before BoNT-A treatment (T0), then at 4 (T1), at 12 (T2), and at 24 weeks (T3) of follow-up. PSS was assessed using the MAS [23]; this 6-point scale grades the resistance of a relaxed limb to rapid passive stretch (0 = no increase in muscle tone; 1 = slight increase in muscle tone manifested by a catch and release or by minimal resistance at the end of the range of motion; 1+ = slight increase in muscle tone manifested by a catch, followed by minimal resistance throughout the remainder—less than half—of the range of motion; 2 = more marked increase in muscle tone through most of the range of motion; 3 = considerable increase in muscle tone, passive movement difficulty; 4 = affected part is rigid). Affected upper-limb muscle groups were tested for the following PSS patterns: adducted shoulder with internal rotation, flexed elbow, pronated forearm, flexed wrist, flexed fingers, thumb-in-palm and clenched fist. Affected lower-limb muscle groups were tested for the following PSS patterns: adducted thigh, flexed knee, extended knee, equinovarus foot, plantar flexed foot/ankle, striatal toe, and flexed toes.

The strength of the affected limbs was assessed using the MI [27]. The upper-limb subscale tests shoulder abduction, elbow flexion, and pinch grip, while the lower-limb subscale tests hip flexion, knee extension, and foot dorsiflexion. Scoring for all movements (except grip) is: 0 = no movement; 9 = palpable contraction in muscle, but no movement; 14 = visible movement, but no full range and not against gravity; 19 = full range of movement against gravity, but no resistance; 25 = full movement against gravity but weaker than the other side; 33 = normal power. Grip scoring is 0 = no movement; 11 = beginning of prehension; 19 = able to grip cube, but not hold it against gravity (examiner may need to lift the wrist); 22 = able to grip and hold the cube against gravity; 26 = able to grip and hold the cube against a weak pull, but weaker than the other side; 33 = normal power. The maximum total score is 99 (+1) for the upper and the lower limb.

The FMA was used to evaluate motor recovery after stroke [28]. The total maximum score is 226; FMA comprises five domains, including motor function (upper-limb maximum score = 66; lower-limb maximum score = 34), sensory function (maximum score = 24), balance (maximum score = 14), joint range of motion (maximum score 44), and joint pain (maximum score = 44).

The degree of disability after stroke was graded according to the MRS as follows: 0 = no symptoms at all; 1 = no significant disability despite symptoms (able to carry out all usual duties and activities); 2 = slight disability (unable to carry out all previous activities, but able to look after own affairs without assistance); 3 = moderate disability (requiring some help, but able to walk without assistance); 4 = moderately severe disability (unable to walk without assistance and unable to attend to own bodily needs without assistance); 5 = severe disability (bedridden, incontinent and requiring constant nursing care and attention); 6 = dead [29].

### 5.1. Statistical Methods

Descriptive statistical analysis relied on frequency tables and bar plots for categorical variables. Boxplots represent continuous variables. Continuous variables were summarized with the average and standard deviation or the median and IQR depending on marginal distribution of the variable. Data were tested for normality using the Shapiro–Wilk test. Student’s *t*-test or the Wilcoxon rank-sum test was computed to compare the distribution across two strata in case of normal or non-normal distribution. Analysis of the variance (ANOVA) or the Kruskal–Wallis test was applied to more than two strata. A Kendall correlation test was performed to evaluate the trend between continuous variables and follow-up phase. The distribution of categorical variables was compared using the Chi-squared test. If the expected frequencies in any combination were <10, we performed Fisher’s exact test. Statistical significance was set at 5%. Statistical analysis was performed using R (https://www.R-project.org/, accessed on 23 May 2021).

#### Cumulative Logistic Mixed-Effects Model

To identify the factors that influenced PSS severity, we took as the dependent variable the MAS score, which assumes integer values between 1 and 4. Given the distribution of selected outcome, we employed a cumulative logistic model as follows:

(1) $$logit\left(P\left(Y_{i}\leq k\right)\right)=\theta_{k}-\left(X_{i}^{T}\beta +\epsilon_{i}\right),$$

where *k* = 0, …, 4 are the possible values of MAS and $Y_{i}$ is the observed MAS value for the *i*-th observation. Parameters are the intercept (which depends on *k*), while β is the vector of coefficients of the regression matrix X. $\epsilon_{i}$ is the regression error at zero mean and constant variance that follows a normal distribution $N\left(0,\sigma_{\epsilon }^{2}\right).$ The MAS score depends on the muscles treated and individual susceptibility. In a hierarchical structure (repeated measurements per muscle group and subject for each follow-up time), we denoted with $Y_{ijt}$ the value of MAS for the *i*-th subject in the muscle group *j* (with *j* = 1,..., *J*) at time *t* (with *t* = 1, ..., 4). The model thus takes the following form:

(2) $$logit\left(P\left(Y_{ijt}\leq k\right)\right)=\theta_{k}-\left(X_{i}^{T}\beta +\gamma_{ijt}+\epsilon_{ijt}\right),$$

where β is the coefficient of the fixed effect. The random effect parameter $\gamma_{ijt}$ is structured as follows $\gamma_{ijt}=\gamma 0_{t}+\varphi 0_{it}+\varphi 1_{jt}$ which is the sum of three random effects: $\gamma 0_{t}\sim N\left(0,\sigma_{\gamma 0}^{2}\right)$ is related to time *t*, and $\varphi 0_{it}\sim N\left(0,\sigma_{\varphi 0}^{2}\right)$ and $\varphi 1_{jt}\sim N\left(0,\sigma_{\varphi 1}^{2}\right)$ to subject-time and muscle group–-time interactions, respectively. Residual error is $\epsilon_{ijt}\sim N\left(0,\sigma_{\epsilon }^{2}\right)$. To account for factors potentially affecting PSS severity, the following fixed effects were included: sex, time between stroke onset and initial BoNT-A injection (<90 days; ≥90 days), follow-up time (*t* = 0,1,2,3), age group at stroke onset (≤60; 60–70; >70 years), and BoNT-A vial dilution in mL. We kept the previous covariates on the basis of Akaike information criterion and forward selection criteria testing second-order interactions. The results were presented using OR by exponentiating the estimated coefficients from the cumulative logistic regression. The model was estimated by R and the clmm package [30].

## Figures and Tables

**Table 1:** Patient characteristics stratified by sex.

	Overall	Women (*n* = 31)	Men (*n* = 52)	*p*-Value
**Study center**, No. (%)				0.58
Center #1	8 (9.6)	2 (6.5)	6 (11.5)	
Center #2	12 (14.5)	6 (19.4)	6 (11.5)	
Center #3	15 (18.1)	3 (9.7)	12 (23.1)	
Center #4	6 (7.2)	3 (9.7)	3 (5.8)	
Center #5	8 (9.6)	3 (9.7)	5 (9.6)	
Center #6	8 (9.6)	3 (9.7)	5 (9.6)	
Center #7	1 (1.2)	1 (3.2)	0 (0.0)	
Center #8	7 (8.4)	4 (12.9)	3 (5.8)	
Center #9	18 (21.7)	6 (19.4)	12 (23.1)	
**Age**, years. Mean (±SD)	63.9 (12.5)	65.5 (12.5)	63.0 (12.5)	0.38
**Time since stroke onset**, Mean (±SD)	136.1 (95.1)	118.4 (86.6)	146.7 (99.1)	0.18
**Type of stroke**, No. (%)				0.87
Hemorrhagic	25 (30.1)	9 (29.0)	16 (30.8)	
Ischemic	58 (69.9)	22 (71.0)	36 (69.2)	
**Lesion side**, No. (%)				0.93
Right	45 (54.2)	17 (54.8)	28 (53.8)	
Left	38 (45.8)	14 (45.2)	24 (46.2)	
**Hospital regimen**, No. (%)				0.56
Outpatient	33 (39.8)	10 (32.3)	23 (44.2)	
Day hospital	10 (12.0)	4 (12.9)	6 (11.5)	
Inpatient	40 (48.2)	17 (54.8)	23 (44.2)	
**Injection technique**, No. (%)				0.56
Ultrasound-guided	54 (65.1)	18 (58.1)	36 (69.2)	
Electrical stimulation	11 (13.3)	5 (16.1)	6 (11.5)	
Manual needle placement	18 (21.7)	8 (25.8)	10 (19.2)	
**Vial dilution**, No. (%)				0.67
1 mL	11 (13.3)	5 (16.1)	6 (11.5)	
1.5 mL	5 (6.0)	3 (9.7)	2 (3.8)	
2 mL	56 (67.5)	19 (61.3)	37 (71.2)	
2.5 mL	11 (13.3)	4 (12.9)	7 (13.5)	

Abbreviations: SD, standard deviation.

**Table 2:** MRC scale grades stratified by muscle group and patient sex.

Muscles	Overall	Women (*n* = 31)	Men (*n* = 52)	*p*-Value *
Shoulder External Rotators, Median (IQR)	1.00 (2.00)	1.00 (2.00)	1.00 (2.00)	0.27
Shoulder Abductors, Median (IQR)	2.00 (2.00)	1.00 (2.00)	2.00 (1.00)	0.20
Elbow Extensors, Median (IQR)	1.00 (2.00)	1.00 (2.00)	2.00 (3.00)	0.04
Elbow Flexors, Median (IQR)	2.00 (2.00)	1.00 (2.00)	2.00 (3.00)	0.06
Forearm Supinators, Median (IQR)	1.00 (2.00)	0.00 (1.00)	1.00 (2.25)	0.08
Wrist Extensors, Median (IQR)	0.00 (2.00)	0.00 (1.00)	1.00 (2.00)	0.08
Finger extensors, Median (IQR)	0.00 (2.00)	0.00 (1.00)	1.00 (2.00)	0.05
Hip Extensors, Median (IQR)	3.00 (1.00)	2.00 (1.00)	3.00 (1.00)	0.14
Hip External Rotators, Median (IQR)	2.00 (1.00)	2.00 (2.00)	3.00 (1.25)	0.13
Hip Abductors, Median (IQR)	2.00 (2.00)	2.00 (1.75)	2.50 (2.00)	0.22
Knee Extensors, Median (IQR)	3.00 (2.00)	3.00 (1.75)	3.00 (2.00)	0.32
Knee Flexors, Median (IQR)	3.00 (2.00)	3.00 (1.00)	3.00 (2.00)	0.33
Ankle Invertors, Median (IQR)	1.00 (3.00)	1.00 (2.00)	2.00 (2.25)	0.16
Ankle Dorsiflexors, Median (IQR)	2.00 (3.00)	1.00 (2.00)	2.00 (2.00)	0.13
Ankle Evertors, Median (IQR)	1.00 (2.00)	0.50 (1.75)	1.50 (2.00)	0.03

* Wilcoxon sum test; Abbreviations: MRC, Medical Research Council; IQR interquartile range.

**Table 3:** FMA score for the Upper Limb (UL) and the Lower Limb (LL) stratified by follow-up time.

	0 Weeks	4 Weeks	12 Weeks	24 Weeks	*p*-Value *
UL, Median (IQR)	8.00 (9.50)	11.50 (15.00)	14.00 (17.00)	16.00 (16.20)	0.001
Wrist, Median (IQR)	0.00 (3.00)	0.00 (2.00)	0.00 (4.00)	0.00 (4.00)	0.023
Hand, Median (IQR)	0.00 (5.00)	0.00 (5.00)	0.00 (7.00)	0.00 (7.00)	0.283
UL Speed coordination, Median (IQR)	1.00 (3.00)	2.00 (3.00)	2.00 (4.00)	2.00 (4.00)	0.045
UL Motor function, Median (IQR)	9.00 (20.50)	12.00 (25.80)	18.00 (28.00)	18.00 (28.00)	0.016
UL Sensation, Median (IQR)	10.00 (5.00)	10.50 (4.00)	10.50 (4.00)	11.00 (4.00)	0.218
UL Passive motility, Median (IQR)	19.00 (7.50)	20.00 (6.00)	20.00 (7.00)	19.50 (7.00)	0.358
UL Pain, Median (IQR)	16.00 (10.50)	20.00 (10.00)	20.00 (8.00)	20.00 (7.00)	0.007
LL, Median (IQR)	13.00 (7.00)	15.00 (8.00)	16.00 (10.50)	16.00 (11.00)	0.002
LL Speed coordination, Median (IQR)	2.00 (3.00)	3.00 (2.00)	3.00 (2.00)	3.00 (2.75)	<0.001
LL Motor function, Median (IQR)	15.00 (9.00)	17.00 (7.00)	18.00 (8.00)	19.00 (10.00)	<0.001
LL Sensation, Median (IQR)	10.00 (6.00)	11.00 (3.00)	11.00 (4.00)	12.00 (3.00)	0.049
LL Passive motility, Median (IQR)	18.00 (4.00)	18.00 (4.00)	18.00 (3.50)	18.00 (3.00)	0.192
LL Pain, Median (IQR)	20.00 (2.00)	20.00 (2.00)	20.00 (0.00)	20.00 (0.80)	0.291

* Kendall trend test; Abbreviations: FMA, Fugl-Meyer Assessment; IQR, interquartile range.

**Table 4:** MAS score stratified by muscle groups and patient sex.

Muscles	Total *n* = 1167 (100%)	Women *n* = 406 (100%)	Men *n* = 761 (100%)	*p*-Value *
Shoulder Adductors	*n* = 76 (6.4%)	*n* = 32 (7.6%)	*n* = 44 (5.7%)	
Elbow Extensors	*n* = 32 (2.7%)	*n* = 0 (0.0%)	*n* = 32 (4.2%)	
Finger Flexors	*n* = 192 (16.2%)	*n* = 68 (16.2%)	*n* = 124 (16.1%)	
Toe Flexors	*n* = 36 (3.0%)	*n* = 16 (3.8%)	*n* = 20 (2.6%)	
Elbow Flexors	*n* = 184 (15.5%)	*n* = *72* (17.1%)	*n* = 112 (14.6%)	
Thumb Flexors	*n* = 80 (6.7%)	*n* = 24 (5.7%)	*n* = 56 (7.3%)	0.003
Wrist Flexors	*n* = 204 (17.2%)	*n* = 68 (16.2%)	*n* = 136 (17.7%)	
Ankle Plantiflexors	*n* = 132 (11.1%)	*n* = 56 (13.3%)	*n* = 76 (9.9%)	
Forearm Pronators	*n* = 112 (9.4%)	*n* = 36 (8.6%)	*n* = 76 (9.9%)	
Ankle Invertors	*n* = 64 (5.4%)	*n* = 24 (5.7%)	*n* = 40 (5.2%)	
Others	*n* = 76 (6.4%)	*n* = 24 (5.7%)	*n* = 52 (6.8%)	

Abbreviations: *n*, number; MAS, Modified Ashworth Scale.

**Table 5:** Odds ratio estimated by the cumulative logistic model for MAS score compared to the reference category *.

Predictors	Odds Ratio	95% CI	*p*-Value
Follow-up (4 weeks)	0.05	0.03–0.09	<0.001
Follow-up (12 weeks)	0.06	0.04–0.11	<0.001
Follow-up (24 weeks)	0.24	0.14–0.41	<0.001
TSO (>90 days)	1.71	0.74–3.98	0.210
Age groups (60,70)	0.73	0.32–1.64	0.444
Age group (70,100)	0.35	0.15–0.80	0.013
Sex (Male)	1.18	0.58–2.40	0.643
Vial dilution (mL)	0.12	0.05–0.30	<0.001
Follow-up (4 weeks) * TSO (>90 days)	2.05	1.02–4.13	0.044
Follow-up (12 weeks) * TSO (>90 days)	2.87	1.46–5.65	0.002
Follow-up (24 weeks) * TSO (>90 days)	1.12	0.56–2.26	0.747
Observations/Subjects	1167/83		
Marginal R^2^/Conditional R^2^	0.266/0.601		

* Follow-up 0 weeks, TSO ≤ 90 days, Female. Abbreviations: MAS, Modified Ashworth Scale; TSO, time between stroke onset and BoNT-A injection; CI, confidence interval.

## Data Availability

The data presented in this study are available on request from the corresponding author.
